# Lipid Peroxidation in Algae Oil: Antagonist Effects of Natural Antioxidants

**DOI:** 10.3390/molecules27144453

**Published:** 2022-07-12

**Authors:** Ilaria Santoro, Anna Russo, Enzo Perri, Giovanni Sindona, Monica Nardi

**Affiliations:** 1 QUASIORA Laboratory, Agrinfra Research Net, Università della Calabria, Via P. Bucci, Cubo 12/D, 87036 Rende, Italy; 2 Council for Agricultural Research and Economics (CREA), Research Centre for Olive, Fruit and Citrus Crops, 87036 Rende, Italy; rsanna75@libero.it (A.R.); enzo.perri@crea.gov.it (E.P.); 3 Department of Chemistry and Chemical Technologies, University of Calabria, 87036 Rende, Italy; giovanni.sindona@unical.it; 4 Department of Health Sciences, University Magna Græcia of Catanzaro, 88100 Catanzaro, Italy; monica.nardi@unicz.it

**Keywords:** algae oil, DHA, EPA, POBN nitrone, tandem mass spectrometry, natural antioxidants

## Abstract

Tandem mass spectrometry is proposed to check lipid oxidation, a free radical-mediated phenomenon which effects oxidative deterioration in polyunsaturated fatty acids. Antioxidants are used by the food industry to delay the oxidation process. This process can be controlled by antioxidants, which may occur as natural constituents of foods or may be intentionally added to products. Synthetic antioxidants such as BHT, BHA, and propyl gallate have been extensively used as antioxidants in the industry. The worldwide tendency to avoid or minimize the use of synthetic food additives has prompted the replacement of synthetic antioxidants with natural analogues. The entire process can be supported by the detection and characterization of the reacting species by suitable application of electrospray tandem mass spectrometry under collision-induced dissociation (ESI-CID-MS/MS). Natural antioxidants were tested in this study to check the oxidative stability of algae oil when adding the natural additive. Results were observed in algae oil in situ using electrospray mass spectrometry in tandem with collision-induced dissociation tandem mass spectrometry (ESI-CID-MS/MS) and the POBN spin trapper. The results indicate that alpha-tocopherol is a better antioxidant.

## 1. Introduction

Consumption of polyunsaturated fatty acids (PUFA) of the omega-3 series exerts a strong positive influence on decreasing the risk factors associated with the occurrence of several degenerative diseases including cancer, cardiovascular diseases, and other inflammatory conditions [1]. These studies provide the rationale for using docosahexaenoic acid (DHA) supplements to enrich the diet of pregnant and lactating women.

Urea complexation of seal blubber oil [2] and of algal oil [3] was attempted for producing ω-3 PUFA concentrates, which is a well-established technique for elimination of saturated and monounsaturated fatty acids. The current interest in using long-chain polyunsaturated oils, including DHA-containing oils, in infant foods [4] demands and has favored the development of valid stability tests and endpoints of quality deterioration [5,6]. In this direction, tandem mass spectrometry coupled with soft ionization methods could be the analytical protocol of choice.

DHA-rich oils from algae were claimed to have an unusually high oxidative stability [7], but the evidence for the basis of this stability may be questionable. Claims for high stability of DHA-rich oils based on the Rancimat method are subject to serious pitfalls [8]. The use of conductivity detection in the Rancimat method is neither sufficiently sensitive nor sufficiently specific for measurement of oxidative decomposition products of n-3 PUFA responsible for undesirable development of degradation processes [9]. The object of this study is the combined application of spin trapper and tandem mass spectrometry techniques in monitoring the evolution in situ of docosahexaenoic acid (DHA) and eicosapentaenoic acid (EPA) radical species in algae oil with and without the assistance of natural antioxidants such as polyphenols present in extra virgin olive oil, tocopherols, etc.

Reactive oxygen species (ROS) have been implicated in the pathogenesis of many diseases, including cancer, mutagenesis, Alzheimer’s, AIDS, etc. Many synthetic antioxidants are currently in use; nevertheless, there is a growing evidence of consumer preference for natural antioxidants because of their potentially lower toxicity. One of the main detrimental effects of reactive radical species (e.g., OH•) is lipid peroxidation (LP), i.e., oxidative degradation of lipids, leading to biological membrane damage and, possibly, to cell death or formation of mutagenic/carcinogenic products [10]. The lipid oxidation in foods is responsible for the formation of off-flavours and undesirable chemical compounds which may be detrimental to health. Antioxidants are used by the food industry to delay the oxidation process. Oils rich in long-chain n-3 polyunsaturated fatty acids (PUFA) have long been recognized for their nutritional importance [11,12,13,14,15,16]. Lipid oxidation, however, limits the utilization of these oils in processed foods and as nutritional supplements in fortified food. This process can be retarded by antioxidants, which may occur as natural constituents of food or be intentionally added to products. Synthetic antioxidants such as BHT, BHA, and propyl gallate have been extensively used as antioxidants in the industry. However, there is an increasing interest in replacing synthetic antioxidants with natural compounds with antioxidative activities because of a worldwide trend to avoid or minimize the use of synthetic food additives.

**Figure 1:**
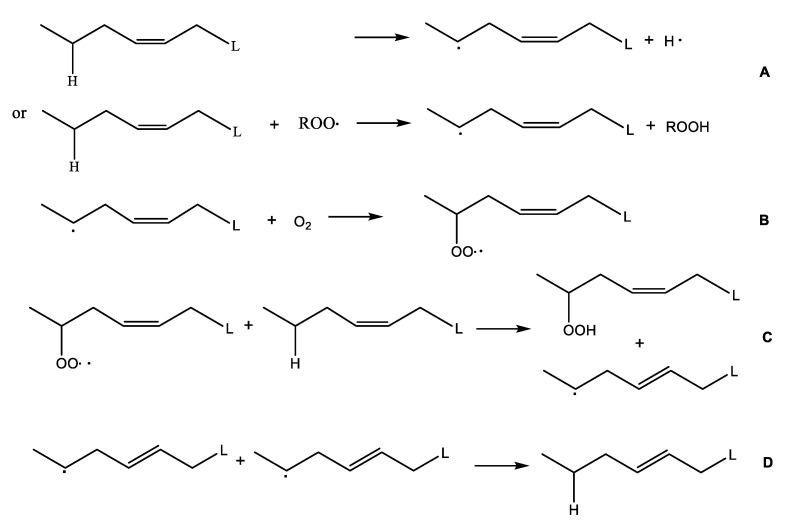
The main lipid peroxidation reactions. Hydrogen is abstracted from an olefinic acid (LH) molecule to form alkyl radicals (L**^.^**), usually in the presence of a catalyst, such as metal ions, light, heat, or irradiation, at a relatively slow rate; alternatively, free peroxide radicals (ROO**^.^**) capture a hydrogen atom from the allylic methylene group conjugated to a double bond in the unsaturated fatty acid chain (LH) with consequent formation of a radical on the carbon atom correspondent (L**^.^**) (**A**), induction. Next, radicals L**^.^** react very quickly with molecular oxygen, generating peroxyl radicals LOO**^.^** (**B**) that subtract a hydrogen atom to another molecule of the unsaturated fatty acid to form a hydroperoxide and a free radical L**^.^** (**C**). The L**^.^** produced react with each other and give rise to products not radicals-inactive (**D**), the termination phase.

The literature on *Schizochytrium* algal strain suggests that it is consumed as food from many invertebrates’ dietary range. In recent years, the strain under examination inspired considerable interest because it is able to produce a total of PUFA equal to 50% of the dry weight of the cell; of this percentage, 30% can be attributed to DHA content [17]. The literature on the oxidative stability of long-chain n-3 PUFA is controversial. This problem may be attributed to the wide variation in fatty acid and triglyceride composition of fish oils [18] and to the wide range of methods and lipid systems used in oxidative stability tests using antioxidants [8] Many different methods have been used to measure the resistance of a lipid to oxidation when in the presence of potential antioxidants. These tests are generally performed in either a lipid or emulsion medium. Autoxidation is a slow radical process [19] which proceeds via a chain reaction including induction, propagation, and termination steps (Figure 1).

The addition of an antioxidant inhibitor (IH) to the system prevents or detects the oxidative propagation chain, stabilizing the generated radical and thus helping to reduce oxidative damage in the human body. The role ascribed to antioxidant molecules concerns their ability to react with reactive radicals to form non-radical products Inactive (NRP), in order to exervise a protective effect against oxidative damage. Most of these tests are performed by speeding up the induction period of the chain reaction with increasing temperature or increased oxygen supply. From these tests, the antioxidative activities of numerous pure compounds and plant extracts have been determined. These methods for oxidation stability evaluation accelerate oxidation under conditions that are too drastic and are unspecific and not sufficiently sensitive to be relevant [20,21,22,23,24,25].

To detect short-lived radicals, the spin-trapping technique was introduced [26,27] and has allowed the detection of many free radicals in biological systems [28] using mass spectrometry to obtain comprehensive knowledge about the structures of the radical adducts.

Over the past several decades, several nitrones have been designed to improve their spin-trapping properties and their biological activities [29]. If used as spin traps, it is important that they have a high free radical entrapment rate constant and a high stability of the corresponding aminoxyl spin adduct to ensure efficient detection.

In previous experiments [30], the adducts formed by α-[4-pyridyl 1-oxide]-N-t-butyl nitrone (POBN) with methyl and methoxy radicals have provided important clues into the radical scavenging role of natural antioxidants in vitro. Subsequently, the antioxidant activity was demonstrated by some amino acids in the spontaneous oxidation of linoleic acid [19], observing a modulating effect when the same amino acids were incubated with soy lipoxygenase and linoleic acid. The elucidation of the reaction pathways was achieved through the identification, by electron spin resonance (ESR) spectroscopy, of POBN-amino acids adducts.

The object of this work is the application of spin-trapper and tandem mass spectrometry techniques [19,31,32,33] in the rapid, sensitive, and unambiguous identification and monitoring of EPA and DHA radical species in algae oil in situ using a radical trapping nitrone as POBN. The method is performed without any manipulation of the conditions and allows polyphenol evaluation in leaves and olive oil (hydroxytyrosol, oleuropein, α-tocopherol), refs. [34,35,36,37,38,39,40,41] phenols evaluation in some distilled alcohol beverages (syringic acid, catechol, caffeic acid), and antioxidant effect or redox-regulators in lipid oxidation. Liquid Chromatography Multiple-Reaction-Monitoring Mass Spectrometry (LC-MRM/MS), a method based on multiple reaction monitoring, is used for the monitoring in the time of formation of a lipid-nitrone adduct and its performance in the presence of natural antioxidants. Adduct formation makes it possible to monitor and study the formation of free radicals after incubation using mass spectrometry. The sample was detected and characterized by electrospray mass spectrometry in tandem with collision-induced dissociation tandem mass spectrometry (ESI-CID-MS/MS) using a POBN spin trapper. Natural antioxidants, added at the same samples, have been useful in controlling algal oil oxidative stability. Next, the lipid profile of the obtained samples was also characterized and determined before and after the oxidative process, showing no variation.

## 2. Results

**Figure 2:**
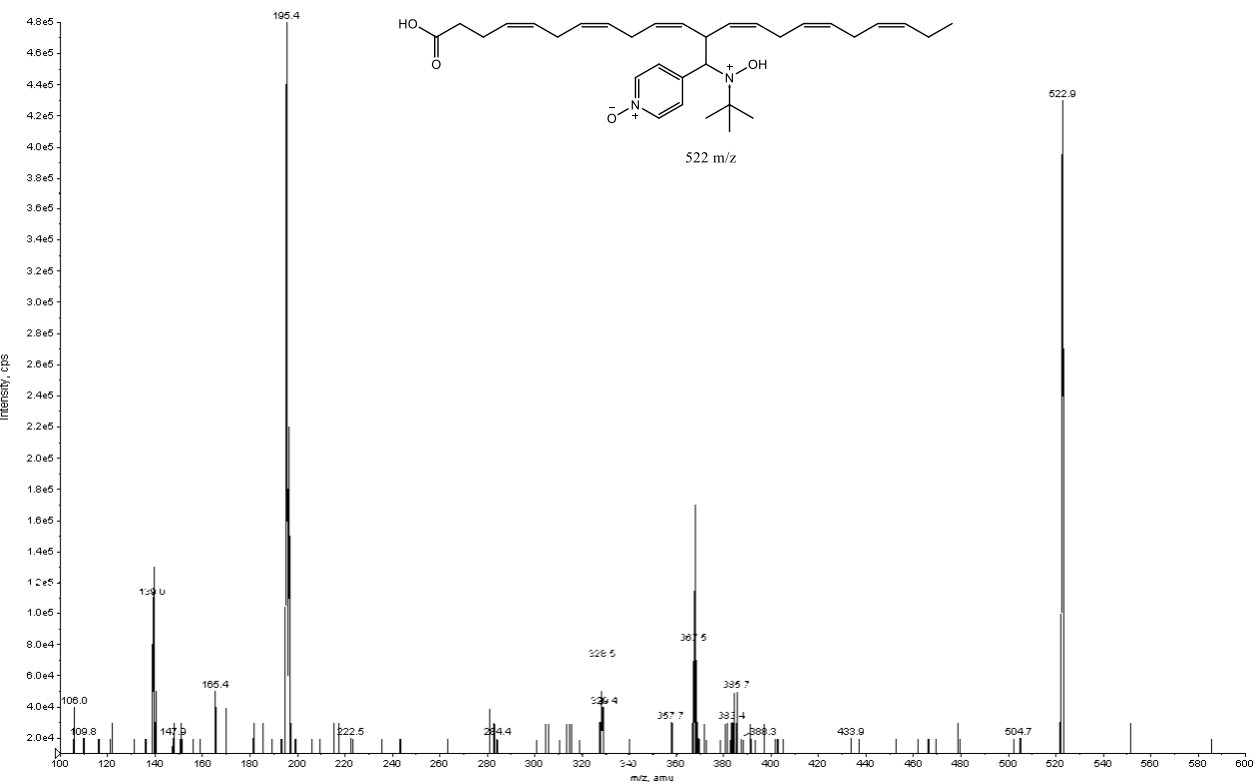
ESI MS/MS spectra and structure of DHA-POBN adduct.

**Figure 3:**
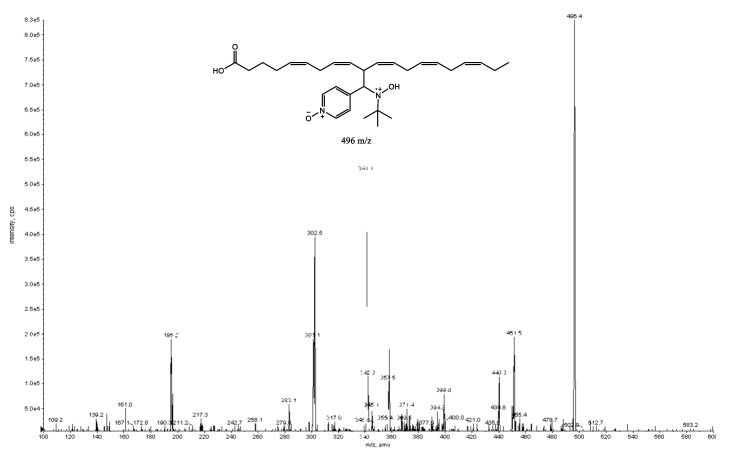
ESI MS/MS spectra and structure of EPA-POBN adduct.

The radical trapping technique has provided insight into the role played by selected natural antioxidants as radical scavengers in the complex lipid oxidation mechanism. At first, the mass spectra of the sample containing the algal oil and the nitrone, after incubation, reveals the presence of unequivocal covalent adducts DHA + POBN + H]^+^ and EPA + POBN + H]^+^ with peaks, respectively, at *m/z* 522 (Figure 2) and *m/z* 496 (Figure 3). Mass spectra of systems containing the authentic standards DHA and EPA instead of algae oil confirm the formation of the adducts, the fragmentation of which is characterized by the loss of the protonated nitrone at *m/z* 195 and of fatty acid (Figure 2).

The tandem mass spectrum is characterized not only by the specific fragments at *m/z* 195 and 328, corresponding, respectively, to the nitrone and the radical cation of the acid, but also by ions *m/z* 367 and *m/z* 386, which establishes that the nitrone could coordinate in position 12 (Figure 2).

**Scheme 1:**
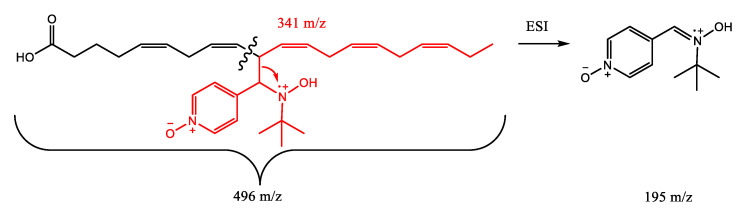
ESI-MS/MS. Fragmentation pattern of the [M + H] + POBN adducts after collisions ESI.

The MS/MS spectra of the EPA-adduct is reported in Figure 3. Of such carboxylic acid in the molecule, eight sites of the methylene carbon are present. The tandem mass spectrum is characterized not only by the specific fragments at *m/z* 195 and 302, corresponding, respectively, to the nitrone and the radical cation of the acid, also by ions *m/z* 341 which establishes that the nitrone could coordinate in position 10 (EPA-adduct molecular fragment, indicated in red in Scheme 1).

**Figure 4:**
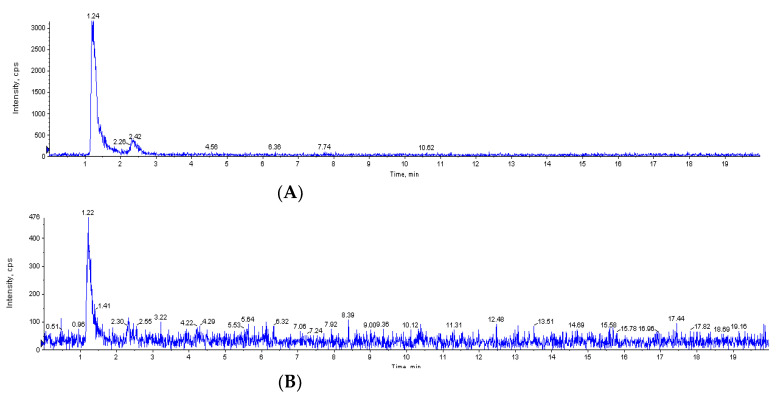
LC-MRM adducts: (**A**) DHA + POBN adduct; (**B**) EPA + POBN adduct.

The monitoring over time of these adducts was carried out a second time, in different systems, each adduct containing algal oil and nitrone in the presence of a natural antioxidant. These results have led us to monitoring in algae oil in liquid chromatography-multiple-reaction monitoring mass spectrometry (LC-MRM/MS) the specific transition PUFA + POBN + H]^+^→POBN + H]^+^ in systems containing the same quantity of natural antioxidants (Figure 4).

Catechol, hydroxytyrosol (HO-Tyr), α-tocopherol, syringic acid, caffeic acid, and oleuropein (Olp) are being used to evaluate their effects on oxidative stability.

**Figure 5:**
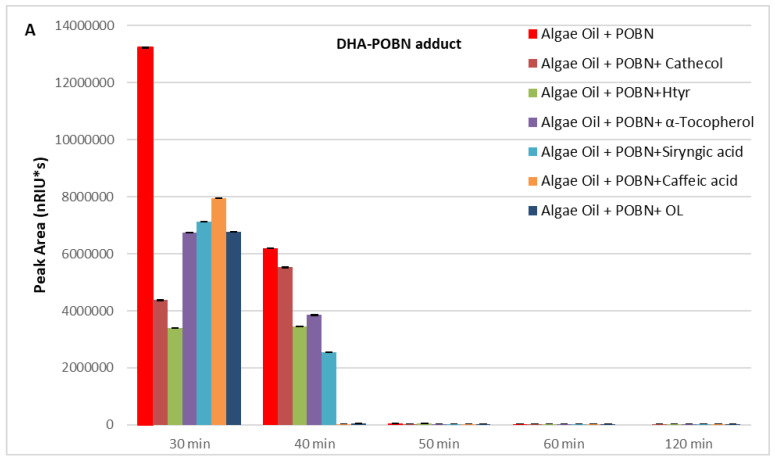

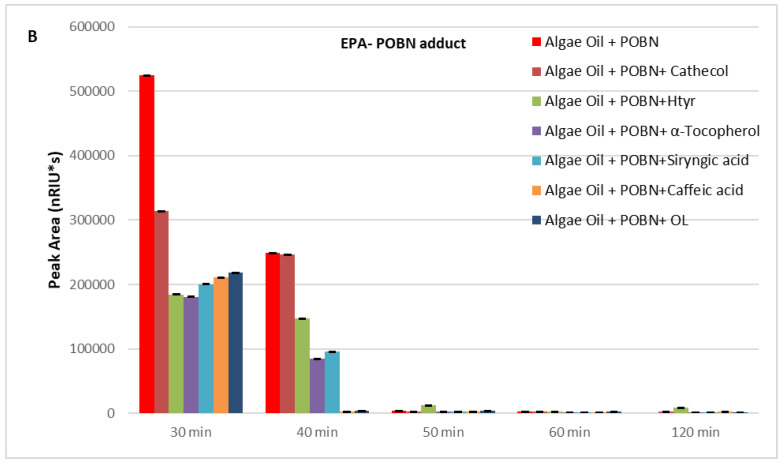
(**A**) Monitoring DHA + POBN adduct; (**B**) Monitoring EPA + POBN adduct.

The monitoring of these adducts in the different times (30, 40, 50, 60 and 120 min) and in the six different systems, after the addition of the six natural antioxidants and then of incubation, reveals a lower content of adducts formed compared to the control system algae oil + POBN (Figure 5). In Figure 5A, the peak area in each of the seven system analyzed (algae oil + POBN; algae oil + POBN + cathecol; algae oil + POBN + HO-Tyr; algae oil + POBN + α-tocopherol; algae oil + POBN + syringic acid; algae oil + POBN + caffeic acid and algae oil + POBN + Olp) corresponds, respectively, to the quantitative monitoring of the DHA + POBN + H]^+^ adduct, following the 328→195 *m/z* MRM transition at different times. Each antioxidant is efficient because in all systems in which it is present, the DHA + POBN + H]^+^ adduct peak area is much lower than the area observed in the control system (red, Figure 5).

In particular, the most effective one seems to be the hydroxytyrosol HT (green). A stable resonance structure is produced by the catecholic structure of HT, which is able to scavenge the peroxyl radicals and interrupt a peroxidative chain reaction [17].

In Figure 5B, the peak area in each of the seven system corresponds, respectively, to the quantitative monitoring of the EPA + POBN + H]^+^ adduct, by following the 496→195 *m/z* MRM transition. In this case, the most effective antioxidant seems to be the α-tochoperol (purple).

As can be seen from the results, mass spectrometry provides very useful answers to satisfy the goal of the present work. The MS spectra of the obtained systems after the incubation of algae oil with different natural antioxidants (NA) demonstrated the presence of DHA and EPA radical provided by the appearance of a protonated adduct with POBN and by the presence of a peak corresponding to the protonated POBN. The MS/MS experiments performed on all the algae oil + POBN + NA samples proved the structure of the spin adducts DHA + POBN and EPA + POBN, whose presence was already suggested by the ESI spectra reported in Figure 1, characterized by the common fragment a *m/z* 195 corresponding to (POBN + H)^+^. The adducts peak area in each of the seven systems analyzed is much lower than the area observed in the control system, demonstrating a substantial activity of natural antioxidants inhibiting the formation of free radicals produced in the processes of lipid peroxidation in algal oil and controlling algal oil oxidative stability.

The results allowed for a clear interpretation and allowed for the spin-trapping technique to monitor the effect that each natural antioxidant tested had on the stability of the algae oil.

To demonstrate that the lipid profile of the samples does not undergo any change, the composition of fatty acids was determined by liquid chromatography (LC)-MS analysis in the samples extracted before and after the oxidative process (See Supporting Information).

## 3. Materials and Methods

### 3.1. Material and Reagents

A commonly used enrichment product, Algamac 3000 series (Aquafuana Bio-Marine Inc, Hawthorne, CA, USA), consists of spray-dried Schizochytrium sp. cells containing 1.30% EPA and 41.30% DHA (*w/w* total fatty acid) based on the manufacturer’s brochure.

POBN (α-[4-pyridyl 1-oxide]-N-t-butyl nitrone), DHA, EPA, catechol, hydroxytyrosol, α-tocopherol, syringic acid, caffeic acid, and oleuropein standards were purchased from Sigma Aldrich (Milano, Italy). HPCL-grade methanol was purchased from Carlo Erba (Cornaredo, Italy). Ultrapure water was obtained from a Milli-Q ultrapure water purification system (Millipore, Burlington, MA, USA).

### 3.2. Algal oil Extraction

A 50 g sample of dried algae was placed in the thimble of the Soxhlet apparatus as described in the literature [42,43].

### 3.3. Samples Preparation

To 100 microliters of alga oil 10 mg of POBN nitrone were added and brought to a volume of 1 mL of acetone. All the samples were incubated for 15 min at 37 °C in a thermostatic bath (Haake D8). The reaction was performed under magnetic stirring at room temperature and monitored in different reaction times: 30, 40, 50, and 120 min, respectively. To verify the results obtained in situ, we prepared similarly two systems containing DHA and EPA (control systems), respectively, instead of oil. Six other systems of algal oil were prepared in situ similarly containing the same fixed quantity (10 mg) of the different natural antioxidants obtaining the following systems to analyze: algae oil + POBN + cathecol, algae oil + POBN + HO-Tyr, algae oil + POBN + α-tocopherol, algae oil + POBN + syringic acid, algae oil + POBN + caffeic acid, and algae oil + POBN + Olp. On each system a 1:100 dilution in 0.1% formic acid in H_2_O and methanol (1/1) was applied before injection into a hybrid mass spectrometer QTRAP API4000 (Applied Biosystem, Waltham, MA, USA). The LC-MRM/MS analysis was repeated after 24, 48, and 72 h of incubation at 25 °C. The spectra did not change with time.

### 3.4. Instrumentation

An Applied Biosystem API 4000 QTRAP hybrid mass spectrometer equipped with an ESI source was used to carry out the analyses. The aqueous part per million solutions of the analytes was delivered to the turbo spray source by direct injection in positive ion mode at a curtain gas (CUR) and source gas (GS1 and GS2) pressure of 20, 20, and 0 PSI, respectively, while the ion spray voltage, the declustering potential, the focusing potential, and the entrance potential were set to 5500, 100, 20 and 10 V, respectively. The tandem mass experiments were performed with a collision energy of 20 V while the CID parameters were set to 2. The LC-MRM analysis of the adducts was carried out using a reversed- phase C18 column. The separation was made using a binary gradient made of H_2_O (O.1% formic acid) (solvent A) and MeOH (solvent B). The gradient is as follows: t = 0 A 90%, t = 5 to 73%, t = 25 A 73%, t = 40 A 30%, t = 43 A 5%, t = 60 A 90%. Each acquisition was repeated three times, and the average result was used in the graphic elaboration discussed in the previous section. Each acquisition was carried out three times, and the average result obtained was used in the graphics processing.

Liquid chromatography (LC)-MS was also a useful method for the accurate analysis of profiling of FFAs using the same parameters as in the previous work [43].

## 4. Conclusions

The natural antioxidants examined in this work have been active in controlling algal oil oxidative stability, to which was added the relationship between the substrate and antioxidant used. A better quantitative understanding of oxidation events and notably of antioxidant reactions in real food would allow a much better prediction of the effects of antioxidants in foods. This knowledge is indispensable for fast, rational design and successful development of functional food products containing non-oxidized n-3 PUFA that are efficiently protected against oxidation. The joint use of the spin trapper and different scanning mode of mass spectrometry in the investigation of radical processes unequivocally allows rapid and sensitive responses which cannot be obtained with other techniques.

Thus, it appears that there are still numerous paths to be explored in the development and application of analytical methods for identification and trace detection of radical species. Furthermore, this study may have great relevance to biology, considering that lipid peroxidation products and modified proteins have been found in human atherosclerotic lesions; however, their pathological significance, as a cause or consequence, has not yet been fully elucidated [44], and not all products of lipid peroxidation are strictly negative for living cells, e.g., 4-hydroxynonenal [45].

## Data Availability

The data presented in this study are available on request from the corresponding author.

## Supplementary Materials

The following supporting information can be downloaded at: https://www.mdpi.com/article/10.3390/molecules27144453/s1, Brief description of experimental methods (mass spectroscopy, extraction techniques) and ESI MS/MS spectra of PUFA-POBN adduct.

## Sample Availability

Samples of the compounds are not available from the authors.
