# Supplementary material for: Lipid Peroxidation in Algae Oil: Antagonist Effects of Natural Antioxidants
Source: Molecules. 2022 Jul 12;27(14):4453. doi: 10.3390/molecules27144453 (PMC9323734; doi:10.3390/molecules27144453)

Lipid Peroxidation in Algae Oil: Antagonist Effects of Natural Antioxidants

Ilaria Santoro [1,*], Anna Russo [2], Enzo Perri [2], Giovanni Sindona [3] and Monica Nardi [4]

[1] QUASIORA Laboratory, Agrinfra Research Net, Università della Calabria, Via P. Bucci, Cubo 12/D, 87036 Rende, Italy
[2] Council for Agricultural Research and Economics (CREA), Research Centre for Olive, Fruit and Citrus Crops, 87036 Rende, Italy; rsanna75@libero.it (A.R.); enzo.perri@crea.gov.it (E.P.)
[3] Department of Chemistry and Chemical Technologies, University of Calabria, 87036 Rende, Italy; giovanni.sindona@unical.it
[4] Department of Health Sciences, University Magna Græcia of Catanzaro, 88100 Catanzaro, Italy; monica.nardi@unicz.it
* Correspondence: ilaria.santoro@unical.it; Tel.: +39-09-8449-3319

Electronic Supplementary Material

Table of Contents

Experimental Section
General Information
Material
Supercritical Fluid Extraction algal oil
ESI MS/MS spectra of PUFA-POBN adduct

Experimental Section

1.1. General Information

An Applied Biosystem API 4000 QTRAP ibrid mass spectrometer equipped with an ESI source was used to carry out analyses. Aqueous part per millions solutions of the analytes were delivered to the turbospray source by direct injection in positive ion mode at a curtain gas (CUR) and source gas (GS1 and GS2) pressure of 20, 20, 0 psi respectively, while the ion spray voltage, the declustering potential, the focusing potential and the entrance potential were set to 5500, 100, 20 and 10 V respectively. The tandem mass experiments were performed with collision energy of 20V while the CID parameters were set to 2. The LC-MRM analysis of the adducts was carried out using a reversed phase C18 column. the separation was made using a binary gradient made of H_2O (O.1% formic acid) (solvent A) and MeOH (solvent B). The gradient is as follows t=0 A 90%; t = 5 to 73%, t = 25 A = 73%, t = 40 A 30%, t = 43 A 5%, t = 60 A 90%. Each acquisition was repeated three times and the average result was used in the graphic elaboration discussed in the previous session. Each acquisition was carried out three times and the average result obtained was used in the graphics processing.
A commonly used enrichment product Algamac 3000 series (Aquafuana Bio-Marine Inc, Hawthorne, CA) consists of spray dried Schizochytrium sp. cells contain 1.30% EPA and 41.30% DHA (w/w total fatty acid) based on the manufacturer's brochure.
POBN (α-[4-pyridyl 1-oxide]-N-t-butyl nitrone), DHA, EPA, catechol, hydroxytyrosol, α-tocopherol, syringic acid, caffeic acid and oleuropein standards were purchased from Sigma Aldrich (Italy). Methanol of HPLC grade was purchased from Carlo Erba (Italy). Distilled water was produced in situ by means of Milli-Q.

1.2. Material

Chemicals Solvents, reagents, and thimbles were purchased from Sigma–Aldrich (Sigma–Aldrich, St. Louis, MO, USA). Dry algal biomass was provided by Aquafauna Biomarine inc. (P.O. Box 5, Hawthorne, CA, USA).

1.3. Soxhlet Extraction algal oil

Soxhlet Extraction was carried out using 100 mL of solvent on 5 g of dried algae sample by Soxhlet apparatus. The extraction lasted for 1 h. The extract was filtered to remove possible solid particles. Organic solutions were then concentrated by rotary evaporation, and the traces of solvent in residual oil were removed by nitrogen flushing. The yield was calculated based on the weight of extracted oil and the weight of the start sample. The used solvent mixture is CPME/EtOH (60:40), best mixture to have a good percental report of SFA:PUFA (saturated fatty acids (SFA) and unsaturated fatty acids (PUFA)) [40. 41]. The extraction was replicated 3 times.

1.4. ESI MS/MS spectra of PUFA-POBN adduct

ESI MS/MS spectra and structure of DHA-POBN adduct

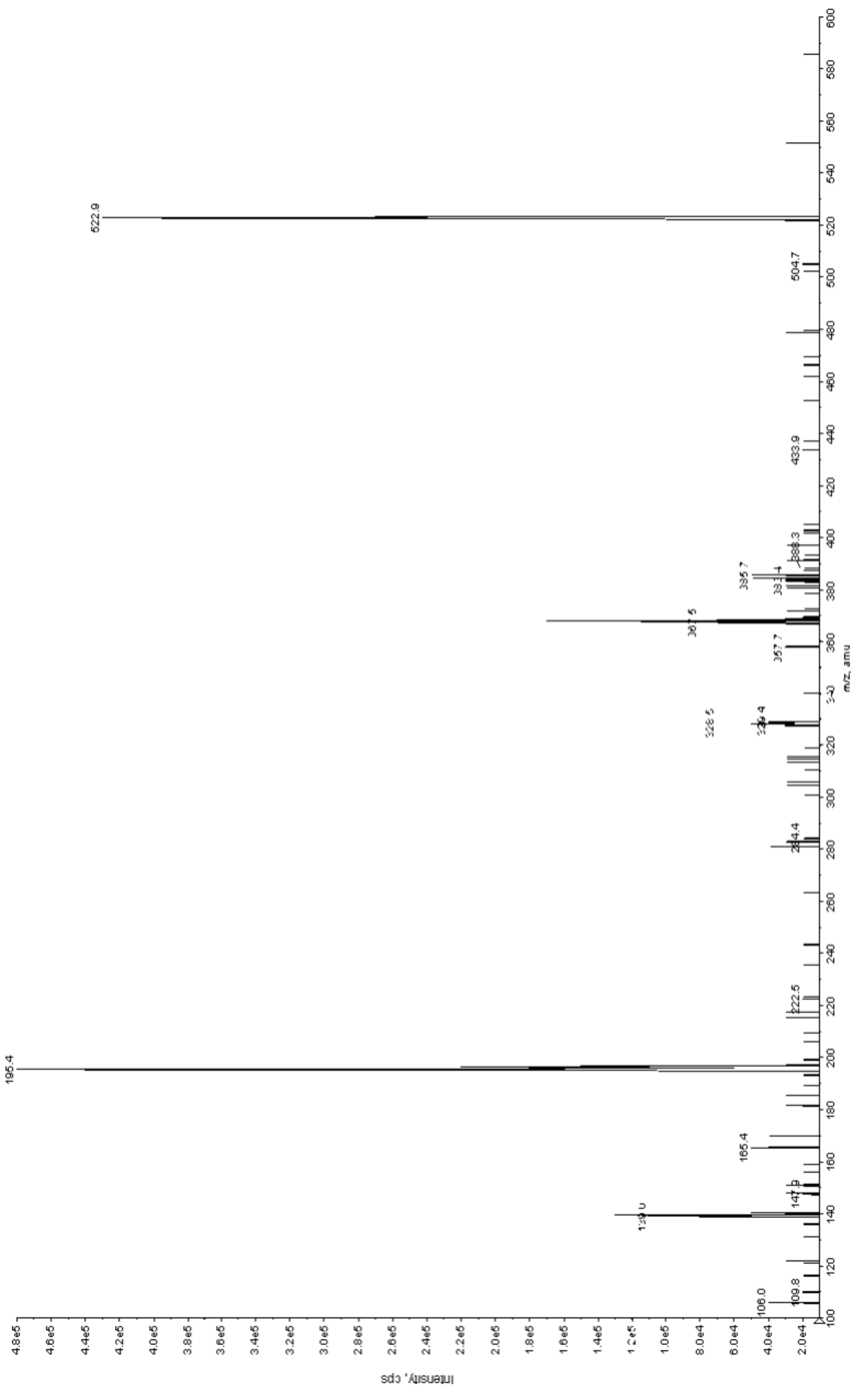

ESI MS/MS spectra and structure of EPA-POBN adduct

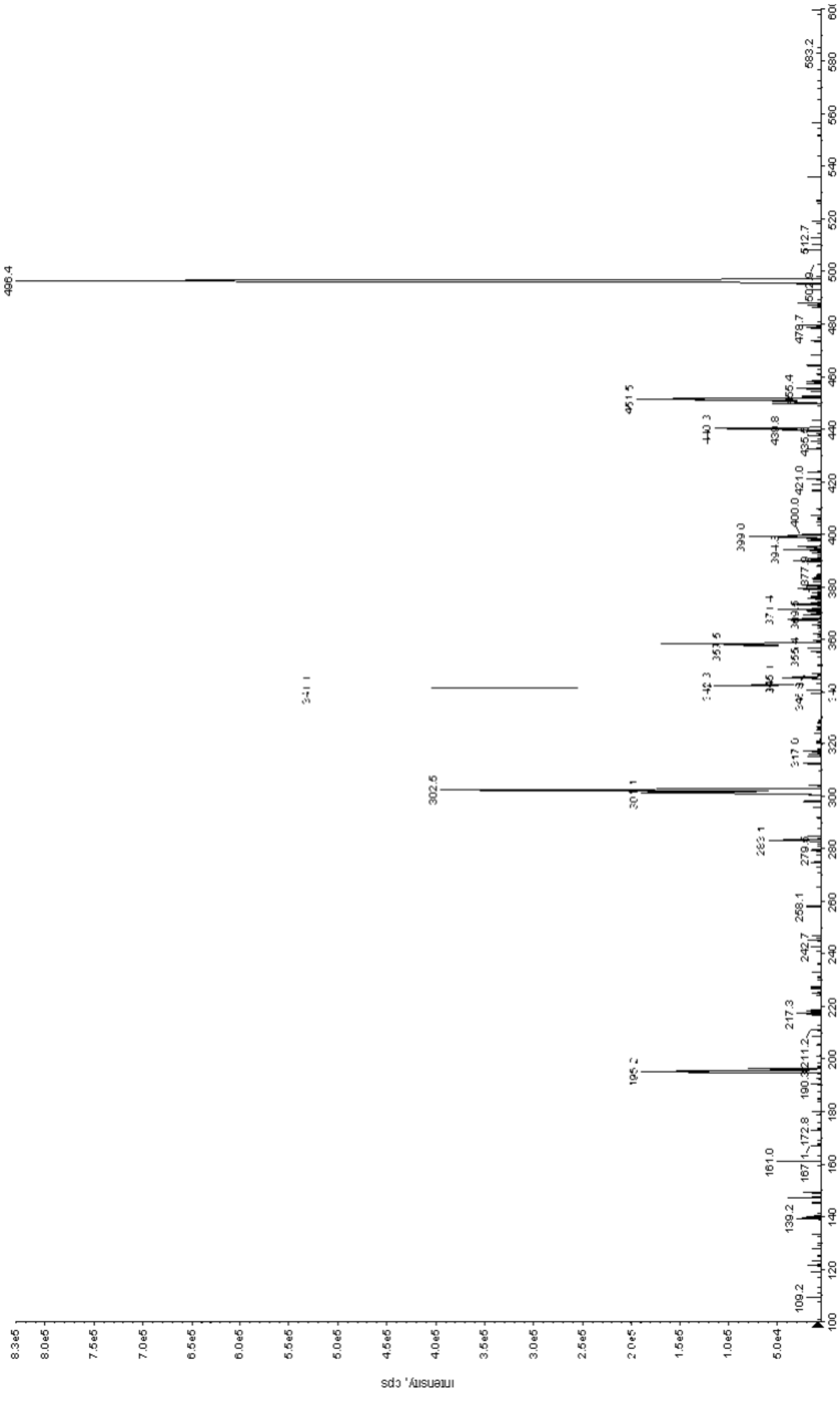

Negative ESI full scan mass spectrum of algal A

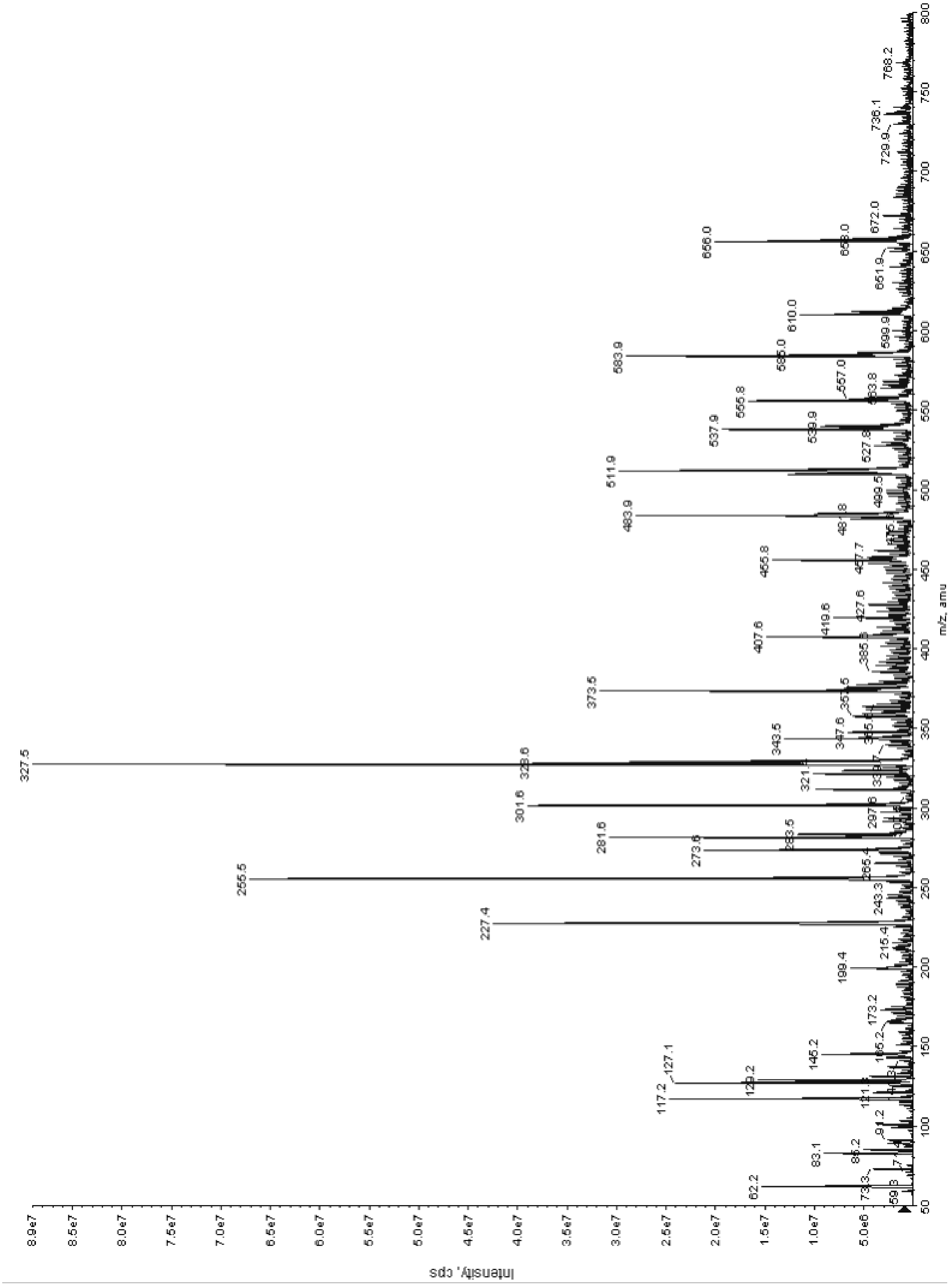

Supplement: Supplementary file 1 [file molecules-27-04453-s001.zip › molecules-1702994-supplementary.pdf]
